# Antidepressant-like activity of sildenafil following acute and subchronic treatment in the forced swim test in mice: effects of restraint stress and monoamine depletion

**DOI:** 10.1007/s11011-016-9852-8

**Published:** 2016-06-10

**Authors:** Katarzyna Socała, Dorota Nieoczym, Mateusz Pieróg, Agnieszka Szuster-Ciesielska, Elżbieta Wyska, Piotr Wlaź

**Affiliations:** 1 Department of Animal Physiology, Institute of Biology and Biochemistry, Maria Curie-Skłodowska University, Akademicka 19, 20-033 Lublin, PL Poland; 2 Department of Virology and Immunology, Institute of Microbiology and Biotechnology, Maria Curie-Skłodowska University, Lublin, Poland; 3 Department of Pharmacokinetics and Physical Pharmacy, Collegium Medicum, Jagiellonian University, Kraków, Poland

**Keywords:** Sildenafil, Depression, Forced swim test, Reserpine, Acute stress, Corticosterone

## Abstract

Sildenafil is a highly effective oral agent for the treatment of erectile dysfunction of multiple etiologies. Although in clinical practice sildenafil is often used in depressed patients, its influence on the pathophysiology of depression remains unclear. The aim of the present study was to evaluate the antidepressant-like activity following acute and subchronic treatment with sildenafil in naïve mice as well as in mice with reserpine- and restraint stress-induced depressive-like behavior. Since corticosterone is released in response to acute stress, we also aimed to assess the influence of sildenafil on serum corticosterone level in non-stressed and stressed animals. The antidepressant activity of sildenafil was assessed in the forced swim test. Corticosterone serum level was determined by using ELISA method, while brain and serum sildenafil level via HPLC method. Sildenafil administered acutely exerted an antidepressant-like effect. Subchronic (14 days) administration of sildenafil resulted only in a weak antidepressant-like effect when evaluated 24 h after the last dose. Acute but not subchronic sildenafil administration reversed the reserpine- and stress-induced immobility in the forced swim test. The lack of effects of sildenafil after subchronic treatment could have been related to its complete elimination from the brain within 24 h from the last injection. Interestingly, acute administration of sildenafil produced a marked increase in serum corticosterone level in both non-stressed and stressed animals. Sildenafil exerts differential effects in the forced swim test after acute and subchronic administration. Further studies on the antidepressant activity of sildenafil are required.

## Introduction

Depression and erectile dysfunction are both highly prevalent disorders that exhibit a marked comorbidity. The causal relationship between them is complex and multifactorial since the occurrence of either disorder may be a symptom or a cause of the other. Moreover, erectile dysfunction is a common adverse effect of antidepressant medication (Nurnberg et al. 2002). Sildenafil citrate, commonly known as Viagra®, is the first-line oral treatment for erectile dysfunction with well-proven efficacy in depressed patients, including those in whom erectile dysfunction developed secondary to antidepressant drug therapy (Nurnberg and Hensley 2003; Seidman et al. 2001). It acts as a selective phosphodiesterase type 5 (PDE5) inhibitor. Following activation of the nitric oxide (NO)/guanosine 3´,5´-monophosphate (cGMP) signaling pathway during sexual arousal, inhibition of PDE5 activity by sildenafil leads to the increase in the intracellular level of cGMP and improves smooth muscles relaxation in the corpus cavernosum (Rosen and McKenna 2002).

Although sildenafil was designed as a drug that works mainly in peripheral tissues, it is capable of crossing the blood brain barrier and affecting central PDE5 activity. Numerous findings showed that sildenafil exerts various beneficial effects in the central nervous system such as stimulation of neurogenesis, memory enhancement, attenuation of the learning impairment and neuroprotection. Not surprisingly, targeting PDE5 with sildenafil has emerged as a new therapeutic strategy in the treatment of many neurological and neuropsychiatric conditions (Uthayathas et al. 2007).

Clinical studies showed that sildenafil is a highly effective oral agent for the treatment of erectile dysfunction in depressed patients (Nurnberg et al. 2002; Nurnberg and Hensley 2003). However, its influence on the symptoms of depression has not been extensively studied in clinics so far. Seidman et al. (2001) reported a marked improvement in depressive symptoms in depressed men with erectile dysfunction treated with sildenafil, but the observed effect was rather due to a significant improvement in the erectile function and a subsequent improvement in quality of life than due to direct mood-enhancing properties of sildenafil. Data obtained in animal studies are contradictory. Several studies showed that sildenafil (at doses ranging up to 20 mg/kg) given acutely does not affect behavioral response of mice challenged with the forced swim test (Almeida et al. 2006; Brocardo et al. 2008; Ghasemi et al. 2008; Savegnago et al. 2008; Socała et al. 2012c). Even though sildenafil given acutely alone had no effect on immobility time in the forced swim test, it was reported to reverse the antidepressant action of many compounds including adenosine (Kaster et al. 2005), memantine (Almeida et al. 2006), diphenyl diselenide (Savegnago et al. 2008), lithium (Ghasemi et al. 2008), escitalopram (Zomkowski et al. 2010), paroxetine (Socała et al. 2012d) or magnesium (Socała et al. 2012a). By contrast, sildenafil augmented the anti-immobility action of some other antidepressant drugs such as amitriptyline (Socała et al. 2012c), mianserin and tianeptine (Socała et al. 2012b).

Brink and co-workers (2008) observed that repeated (7 days) administration of sildenafil (10 mg/kg) did not change immobility time in the forced swim test in rats. Interestingly, in the same study sildenafil exerted antidepressant-like effect after central muscarinic receptors blockage with atropine, which suggests that it may possess antidepressant properties that are attenuated because of its simultaneous cholinotropic action. This observation was further supported by the study, in which we demonstrated that the anti-immobility action of sildenafil (1.25–5 mg/kg) in mice can be revealed even after single administration with joint injection of antimuscarinic agent – scopolamine (Socała et al. 2012c).

It has been subsequently shown that sildenafil possesses acute antidepressant-like effect in the forced swim test at higher doses of 30 and 60 mg/kg. Sub-acute treatment (3 days) with sildenafil at 20 mg/kg also reduced the duration of immobility (Matsushita et al. 2012). Furthermore, a low dose of sildenafil 5 mg/kg was effective against lipopolysaccharide (LPS)-induced depressive-like behavior in mice. This study proved the involvement of the NO/cGMP/PDE5 pathway in the behavioral alterations triggered by LPS injection in mice (Tomaz et al. 2014). It is widely known that inflammation underlies depressive disorder and the anti-inflammatory properties of sildenafil (Raposo et al. 2013; Karakoyun et al. 2011) could have contributed to its antidepressant effect in LPS-treated mice. In another study, Wang et al. (2014) demonstrated that sildenafil given chronically at a dose of 30 mg/kg reversed the depressive-like behavior in the forced swim test as well as in the tail suspension test in mice subjected to the chronic unpredictable mild stress procedure. The observed effect was mediated via the cAMP response element-binding protein (CREB)/brain-derived neurotrophic factor (BDNF)/neuropeptide VGF signaling pathway.

With this background, the present study was undertaken to evaluate the effect of an acute and subchronic treatment with sildenafil in the forced swim test in (a) naïve mice, (b) mice pretreated with reserpine, and (c) mice subjected to acute restraint stress. Reserpine non-selectively depletes brain monoamines and thereby produces a depressive-like behavior in rodents, while acute restraint stress is an unavoidable stress exposure that also causes behavioral despair in animals. Since corticosterone is released in response to stress situation, we aimed also to evaluate the influence of acute and repeated sildenafil treatment on serum corticosterone level in non-stressed and stressed animals. In addition, brain and serum concentrations of sildenafil after acute and subchronic treatment were determined.

## Materials and methods

### Animals

Naïve male Albino Swiss mice weighing 25–30 g were used in the study. The animals were obtained from a licensed breeder (Laboratory Animals Breeding, Słaboszów, Poland) and housed under strictly controlled laboratory conditions (temperature maintained at 22–23 °C, relative humidity 45–55 %) with an artificial 12-h light/dark cycle (light on at 6:00 a.m.). A nutritionally-balanced rodent chow diet (Agropol S.J., Motycz, Poland) and tap water were continuously available. Before being used in the experiments, mice were allowed an adaptation period of at least 7 days. All experiments were performed between 8:00 a.m. and 14:00 p.m. to minimize circadian influences, after a minimum 30-min acclimatization to the experimental room. The animals were randomly assigned to the experimental groups. Each animal was used only once.

The study was carried out under experimental protocols approved by the Ethical Committee of the Medical University in Lublin. All procedures were in strict compliance with the European Union Directive of 22 September 2010 (2010/63/EU) and Polish legislation concerning animal experimentation. All efforts were made to minimize animal suffering as well as the number of animals used in the study.

### Treatments

Sildenafil citrate (kindly provided by Polpharma S.A., Starogard Gdański, Poland) was suspended in a 1 % solution of Tween 80 (POCH, Gliwice, Poland) in normal saline and administered acutely 30 min before the test or repeatedly every 24 h for 14 consecutive days. In the subchronic studies, all experiments were performed 24 h after the last injection in order to avoid acute effects of the drug on animal behavior (Dadomo et al. 2013).

Reserpine was dissolved in a 1 % solution of dimethyl sulfoxide (DMSO, ICN Biomedicals, Inc., Aurora, OH, USA) in normal saline and injected 240 min before challenging animals to the behavioral tests. The procedure of reserpine-induced behavioral despair in mice was conducted according to the method described by Dhir and Kulkarni (2007).

All solutions and suspensions were prepared freshly and administered intraperitoneally (i.p.) in a constant volume of 0.1 ml per 10 g body weight. Control animals received an i.p. injection of a respective vehicle or combination of vehicles.

### Acute restraint stress procedure

Acute restraint stress procedure was performed according to the modified method described by Poleszak et al. (2006). Mice were placed individually in air-assessable Plexiglas cylinders (28 mm diameter, 100 mm long) for 180 min. The animals were not physically compressed during restraint procedure and did not experience pain. They could rotate from a supine to prone position. Immediately after restraint, the animals were moved back to the home cages and challenged to the behavioral tests or decapitation procedure 30 min after immobilization. The non-stressed control animals were left undisturbed in their home cages.

### Forced swim test

The forced swim procedure was carried out according to the slightly modified method of Porsolt et al. (1977). Mice were placed individually into glass cylinders (height 25 cm, diameter 10 cm) containing 11 cm of water maintained at temperature 23–25 °C. Animals were allowed to swim for 6 min. After the initial 2 min of vigorous activity, the total duration of immobility was recorded during the last 4 min of the test. Mice were considered immobile when they stopped struggling, remained floating passively, made no attempts to escape and showed only slow limb movements necessary to keep its head above the water. Water in the beakers was regularly changed between subjects. The immobility time was recorded by a trained observer with the help of cumulative stopwatches. Data obtained in groups of 10–12 mice were expressed as means (in s) ± the standard error of the mean (SEM).

### Locomotor activity test

Spontaneous locomotor activity of mice was measured with the usage of the IR Actimeter system (Panlab/Harvard Apparatus, Barcelona, Spain). The apparatus consisted of a square arena surrounded by a 25 × 25 cm frame containing a total of 16 × 16 infrared beams located on the sides. The frame was coupled to a computerized control unit. Mice were placed individually in the actimeter, in which they were allowed to explore freely for 6 min. The arena was cleaned thoroughly with a 0.1 % acetic acid solution before each mouse was placed in it. Occlusions of the photo beams were recorded automatically and analyzed with a computerized system (SedaCom32, Panlab/Harvard Apparatus, Barcelona, Spain). Locomotor activity was defined as a horizontal activity with displacement and was expressed in terms of total number of interruptions of the photo beams measured during the last 4 min of the test, which corresponds with the time interval analyzed in the forced swim test. Data obtained in groups of 9–12 animals were expressed as means of activity counts/4 min ± SEM.

### Sildenafil determination

Sildenafil was administered at the highest dose used in behavioral studies, i.e., 60 mg/kg. In acute studies, sildenafil was injected 30 min before the decapitation. In subchronic studies, sildenafil was injected for 14 consecutive days and the decapitation was performed 24 h after the last injection.

Concentrations of sildenafil in murine serum and brain tissue were determined according to the method described elsewhere (Strach et al. 2015) with slight modifications*.* Prior to the analysis, brains were homogenized in distilled water (1:4, *w*/*v*) with a tissue homogenizer TH220 (Omni International, Inc., Warrenton, VA, USA). Plasma (50 or 200 μl for acute or subchronic dosing, respectively) or brain homogenate (100 μl or 1 ml) were spiked with 10 μl of 10 μg/ml paroxetine (IS) and vortexed (Reax top, Heidolph, Germany) for 30 s. The samples were alkalized with 50 μl of 4 M sodium hydroxide solution, mixed briefly on the vortex mixer and extracted with 1 ml of ethyl acetate:hexane (30:70, *v*/v) mixture for 20 min on a shaker (VXR Vibrax, IKA, Germany). After centrifugation (EBA 12 R, Hettich, Germany) at 3000 rpm for 15 min, the organic layers were transferred into new Eppendorf tubes containing 100 μl of a methanol and 0.1 M sulfuric acid mixture (10:90, *v*/v). Then the samples were shaken and centrifuged again. Finally, 50 μl of each acidic layer were injected into the HPLC system.

The HPLC system (Thermo Separation Products, San Jose, CA, USA) consisted of a P100 pump fitted with a Rheodyne 7125 manual injector (Cotati, CA, USA) with a 50 μl sample loop, a UV100 variable length UV/VIS detector operating at 230 nm, and an SP4400 integrator (ChromJet). The chromatographic analysis was carried out at 21 °C on the Supelcosil™ PCN column 250x4.6 mm (Sigma-Aldrich, USA) with 5 μm particles, protected with the SUPELCOSIL™ LC-PCN guard column (Sigma-Aldrich, USA) with the same packing material. The mobile phase consisted of acetonitrile and 50 mM potassium dihydrogen phosphate buffer (pH 4.5) mixed in 25:75 (*v*/v) ratio. The flow rate of 1 ml/min was used throughout the analytical run.

The retention times of sildenafil and IS were approximately 7.35 and 9.15 min. The calibration curve constructed by plotting the ratio of the peak area of sildenafil to IS versus concentration of sildenafil was linear in the tested concentration ranges and the limit of quantification was 10 ng/ml (or g tissue). No interfering peaks were observed in the chromatograms. The assay was reproducible with low intra- and inter-day variation (coefficient of variation less than 10 %). The extraction efficiencies of sildenafil and IS were higher than 80 %. Sildenafil concentrations were expressed in μg/ml of serum or μg/g of wet brain tissue.

### Corticosterone determination

In order to determine the influence of sildenafil on serum corticosterone level, the animals were divided into the following groups: (a) non-stressed control animals, (b) stressed control animals, (c) non-stressed sildenafil-treated animals and (d) stressed sildenafil-treated animals. Sildenafil was administered at the highest dose used in behavioral studies, i.e., 60 mg/kg. Sildenafil or vehicle were injected acutely, 30 min before the decapitation. In subchronic studies, sildenafil or vehicle were injected for 14 consecutive days and the decapitation was performed 24 h after the last injection. The trunk blood was collected into polyethylene tubes. Serum was isolated 1 h after blood coagulation by centrifugation at 5000 × *g* for 10 min at 4 °C and frozen at −20 °C. Serum corticosterone level was measured using a commercially available enzyme-linked immunoassay (ELISA) kit (Enzo Life Sciences, Inc., USA; Cat No ADI-900-097) according to the manufacturer’s protocol. Serum corticosterone level was expressed in ng/ml. The minimum detectable concentration of corticosterone was 27.0 pg/ml.

### Statistical analysis

Statistical analysis was performed by using one-way analysis of variance (one-way ANOVA) followed by Tukey *post hoc* test for multiple comparisons. Changes in serum corticosterone level were analyzed by using a two-way ANOVA followed by Bonferroni *post hoc* test. The factors of variation were sildenafil treatment and acute restraint stress exposure. Differences were considered statistically significant when *p* values were equal to or less than 0.05. All calculations were carried out with GraphPad Prism version 5.03 for Windows (GraphPad Software, San Diego, CA, USA).

## Results

### Effect of acute and subchronic sildenafil treatment in the forced swim test in naïve mice

**Fig. 1:**
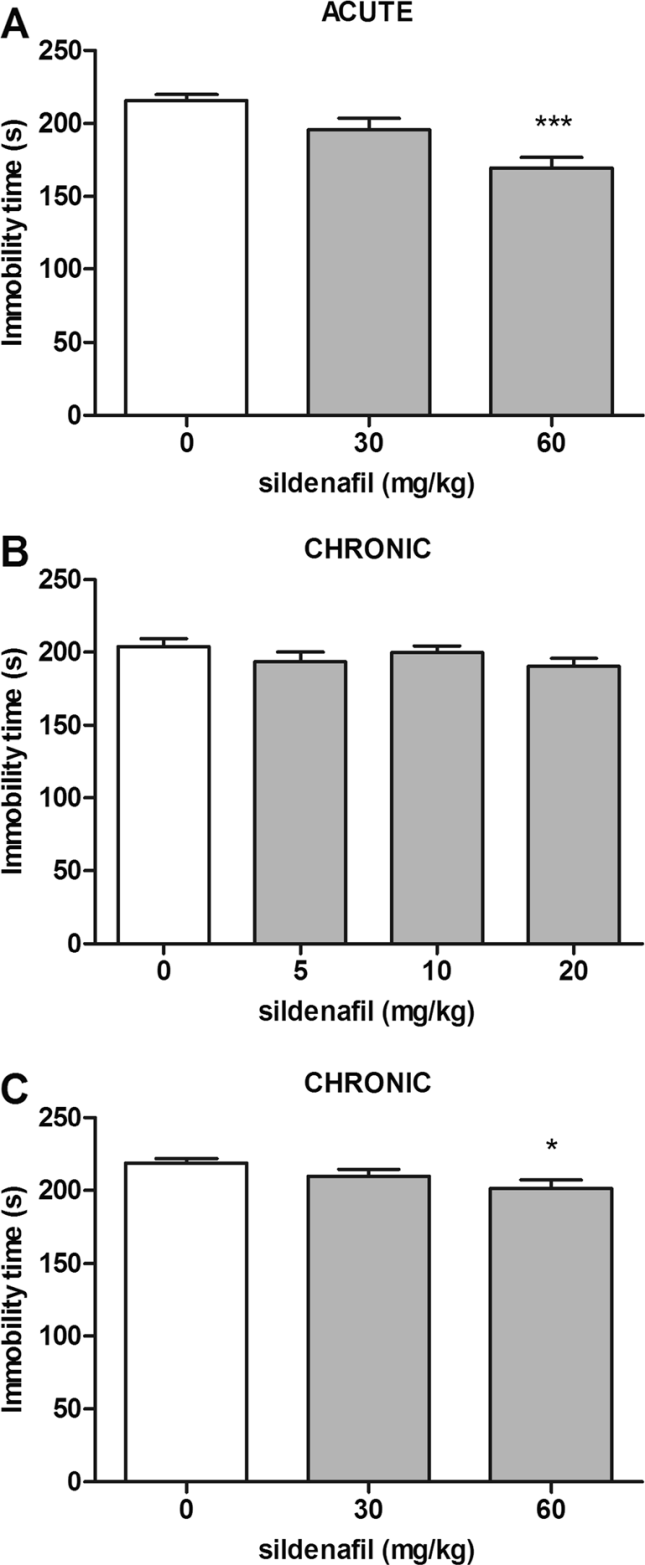
Effect of acute (panel **A**) and subchronic (panel **B** and **C**) treatment with sildenafil in the forced swim test in mice. In acute studies, sildenafil was administered i.p. 30 min before the test. In subchronic studies, sildenafil was administered once daily for 14 consecutive days. Control animals received 1 % Tween 80. Each experimental group consisted of 11–12 animals. Data are presented as means + SEM. * *p* < 0.05, *** *p* < 0.001 as compared to the control group (one-way ANOVA followed by Tukey *post hoc* test)

The effects of acute and subchronic treatments with sildenafil on immobility time in naïve mice is shown in Fig. 1A–C (one way ANOVA: F(2,33) = 12.47, *p* < 0.0001 for acute treatment; F(3,43) = 1.197, *p* = 0.322 and F(2,33) = 3.532, *p* = 0.041 for subchronic treatment, respectively). The first experiment was conducted in order to evaluate the acute antidepressant-like effect of high doses (30 and 60 mg/kg) of sildenafil. The obtained results showed that sildenafil at the highest dose tested, i.e., 60 mg/kg, significantly decreased immobility time as compared to the control group (*p* < 0.001). Repeated administration of sildenafil in a range of doses 5–30 mg/kg did not significantly alter the total immobility duration in the forced swim test in naïve mice. The highest dose tested, i.e., 60 mg/kg, caused only weak anti-immobility effect (*p* < 0.05 vs. the control group).

There were no signs of toxicity or otherwise abnormal behavior following high-dose sildenafil in both treatment regimens.

### Effect of acute and subchronic sildenafil treatment in the forced swim test in reserpine-treated mice

**Fig. 2:**
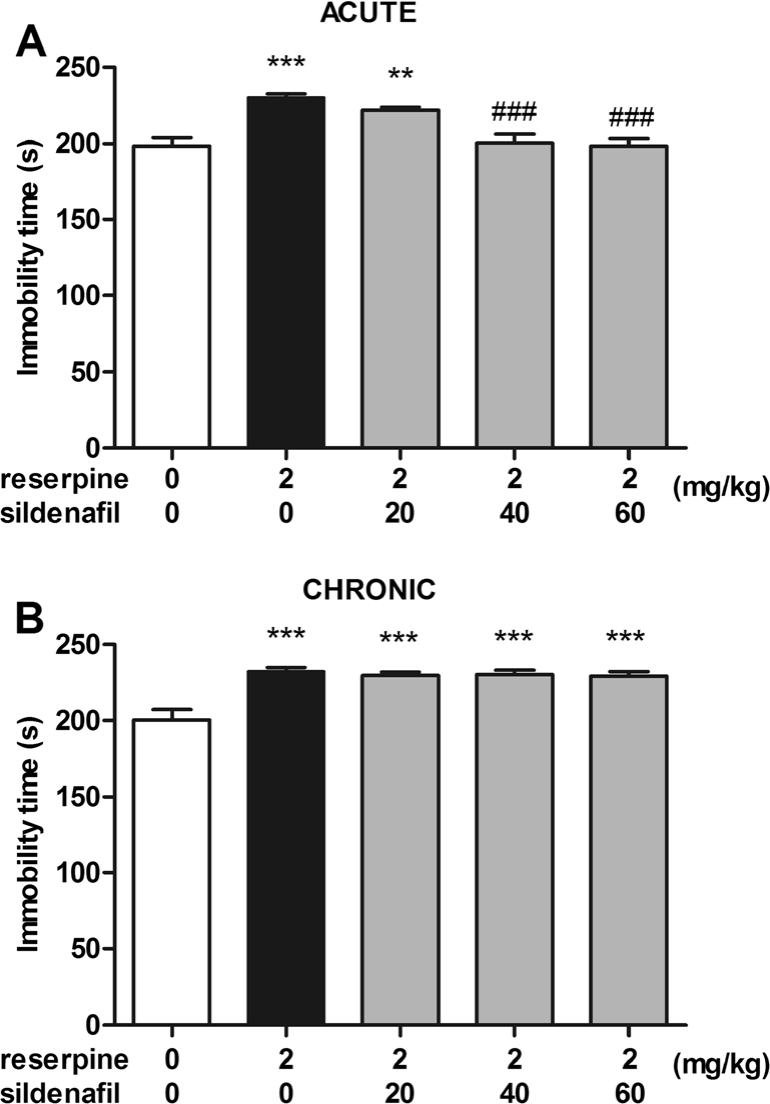
Effect of acute (panel **A**) and subchronic (panel **B**) treatment with sildenafil in the forced swim test in reserpine-treated mice. In acute studies, sildenafil was administered i.p. 30 min before the test. In subchronic studies, sildenafil was administered once daily for 14 consecutive days. Reserpine was injected i.p. 240 min before the test. Control animals received 1 % Tween 80 + 1 % DMSO. Each experimental group consisted of 11–12 animals. Data are presented as means + SEM. ** *p* < 0.01, *** *p* < 0.001 as compared to the control group; ^###^ *p* < 0.001 as compared to the reserpine-treated group (one-way ANOVA followed by Tukey *post hoc* test)

The effects of acute and subchronic administration of sildenafil on immobility time in reserpine-treated mice are shown in Fig. 2A–B (one way ANOVA: F(4,54) = 11.58, *p* < 0.0001 for acute treatment and F(4,54) = 11.64, *p* < 0.0001 for subchronic treatment, respectively). Reserpine (2 mg/kg) produced behavioral despair in mice, which was shown by an increase in the total immobility duration in the forced swim test (*p* < 0.001 vs. control groups in both acute and subchronic studies). Sildenafil injected acutely at doses of 40 and 60 mg/kg reversed the reserpine-induced behavioral despair in mice and caused a significant decrease in the immobility duration as compared to the reserpine-treated group (*p* < 0.001 for both studied groups). However, no such effect was observed when sildenafil, at doses of 20, 40, and 60 mg/kg, was administered repeatedly.

### Effect of acute and subchronic sildenafil treatment in the forced swim test in restraint-stressed mice

**Fig. 3:**
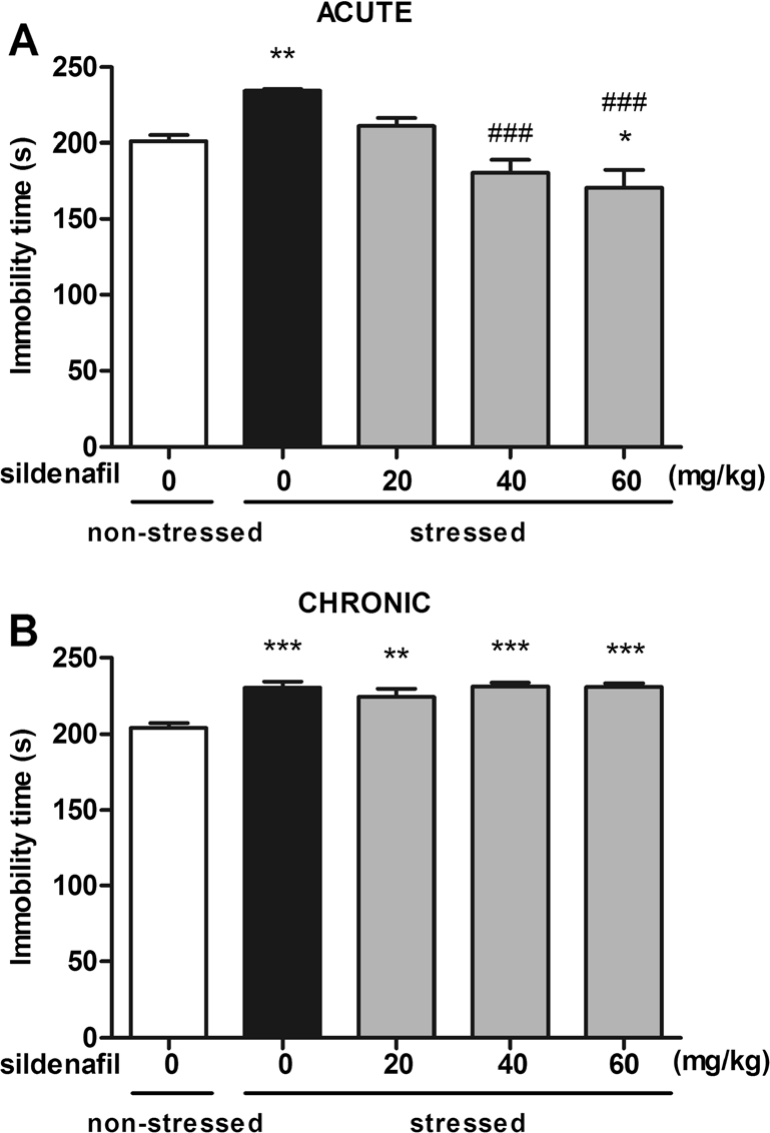
Effect of acute (panel **A**) and subchronic (panel **B**) treatment with sildenafil in the forced swim test in mice subjected to the acute restraint stress. In acute studies, sildenafil was administered i.p. 30 min before the test. In subchronic studies, sildenafil was administered once daily for 14 consecutive days. Control animals received 1 % Tween 80 + 1 % DMSO. Each experimental group consisted of 10–12 animals. Data are presented as means + SEM. * *p* < 0.05, ** *p* < 0.01, *** *p* < 0.001 as compared to the control group; ^###^ *p* < 0.001 as compared to the stressed control group (one-way ANOVA followed by Tukey *post hoc* test)

The effects of acute and subchronic administration of sildenafil on immobility time in mice subjected to the acute restraint stress are shown in Fig. 3A–B (one way ANOVA: F(4,53) = 13.28, *p* < 0.0001 for acute treatment and F(4,52) = 10.98, *p* < 0.0001 for subchronic treatment, respectively). Acute restraint stress induced a significant increase in immobility time in the forced swim test in both acute and subchronic studies (*p* < 0.01 and *p* < 0.001 vs. vehicle-treated non-stressed control mice, respectively). After acute administration, sildenafil at a dose of 20 mg/kg did not produce any significant changes in immobility time as compared to vehicle-treated stressed and non-stressed animals. However, when injected at a dose of 40 mg/kg, sildenafil normalized the increased immobility time in animals subjected to the acute restraint stress (*p* < 0.001 vs. stressed control mice). Sildenafil at the highest dose of 60 mg/kg caused further decrease in immobility time as compared to both vehicle-treated stressed- and non-stressed animals (*p* < 0.001 and *p* < 0.05, respectively). Repeated administration of sildenafil did not affect significantly immobility time as compared to control mice subjected to the acute restraint stress.

### Effect of treatments on spontaneous locomotor activity in mice

As shown in Table 1, no significant changes in locomotor activity were observed following either acute or subchronic administration of sildenafil at doses of 30 and 60 mg/kg (one way ANOVA: F(2,27) = 1.52, *p* = 0.237 for acute treatment and F(2,32) = 1.04, *p* = 0.366 for subchronic treatment, respectively).

**Table 1:** Effect of different treatments on spontaneous locomotor activity in mice

Treatment		Activity counts/4 min	*n*	*p* value
*acute*	1 % Tween 80	1209.0 ± 92.6	10	
	sildenafil 30 mg/kg	974.9 ± 107.0	11	0.237
	sildenafil 60 mg/kg	972.7 ± 132.0	9	
*subchronic*	1 % Tween 80	1191.0 ± 46.6	11	
	sildenafil 30 mg/kg	1283.0 ± 66.2	12	0.366
	sildenafil 60 mg/kg	1324.0 ± 77.4	12	
*acute*	1 % Tween 80 + 1 % DMSO	1083.0 ± 101.5	10	
	1 % Tween 80 + reserpine (2 mg/kg)	77.1 ± 21.1	12	< 0.001
	sildenafil 60 mg/kg + reserpine (2 mg/kg)	65.67 ± 22.7	12	< 0.001
*subchronic*	1 % Tween 80 + 1 % DMSO	1192.0 ± 94.9	12	
	1 % Tween 80 + reserpine (2 mg/kg)	220.8 ± 73.5	12	< 0.001
	sildenafil 60 mg/kg + reserpine (2 mg/kg)	360.1 ± 63.6	12	< 0.001
*acute*	1 % Tween 80	1134.0 ± 88.6	12	
	1 % Tween 80 + restraint stress	786.3 ± 64.0	11	< 0.01
	sildenafil 60 mg/kg + restraint stress	708.4 ± 73.7	11	< 0.01
*subchronic*	1 % Tween 80	963 ± 64.9	12	
	1 % Tween 80 + restraint stress	968.3 ± 94.7	12	0.315
	sildenafil 60 mg/kg + restraint stress	1126 ± 91.5	12	

Data are presented as means ± SEM. In acute studies, sildenafil was injected 30 min before the test. In subchronic studies, sildenafil was administered once daily for 14 consecutive days and the locomotor activity test was performed 24 h after the last injection. Data were analyzed with one-way ANOVA followed by Tukey post hoc test

Reserpine injected alone produced a marked decrease in locomotor activity (*p* < 0.0001 vs. control groups in both acute and subchronic studies). Co-administration of sildenafil at a dose of 60 mg/kg did not cause any additional changes as compared to the reserpine-treated group (one way ANOVA: F(2,31) = 106.2, *p* < 0.0001 for acute treatment and F(2,33) = 44.85, *p* < 0.0001 for subchronic treatment, respectively).

In acute studies, acute restraint stress caused significant decrease in activity counts in the vehicle-treated group (one way ANOVA: F(2,31) = 8.886, *p* = 0.0009). Sildenafil injected at a dose of 60 mg/kg did not cause any additional changes in locomotor activity (*p* < 0.01 vs. the control group). In subchronic studies, no significant changes in locomotor activity were observed (one way ANOVA: F(2,33) = 1.197, *p* = 0.315).

### Brain and serum concentrations of sildenafil

Total brain and serum concentrations of sildenafil after acute and repeated administration (at a dose of 60 mg/kg) are shown in Table 2. Concentrations of this drug in serum and brain tissue measured 30 min following drug administration were comparable and relatively high. This indicates that sildenafil is rapidly and effectively distributed to its site of action. In turn, 24 h after discontinuation of the 14-day subchronic treatment with this drug it was not detectable in brain tissue. In serum, sildenafil was still present 24 h after the termination of the repeated dosing, probably as a result of a slow return of the drug from peripheral tissues to the bloodstream.

**Table 2:** Brain and serum concentrations of sildenafil following different dosing modes

Treatment		Brain concentration (μg/g)	Serum concentration (μg/ml)	*n*
*acute*	sildenafil 60 mg/kg	9.72 ± 1.07	10.98 ± 0.94	12
*subchronic*	sildenafil 60 mg/kg	*not detectable*	0.04 ± 0.01	12

Data are presented as means ± SEM. In acute treatment, sildenafil was administered at a dose of 60 mg/kg, 30 min before decapitation. In chronic treatment, sildenafil was injected for 14 days at a dose of 60 mg/kg and decapitation was performed 24 h after the last injection

### Corticosterone concentrations

**Fig. 4:**
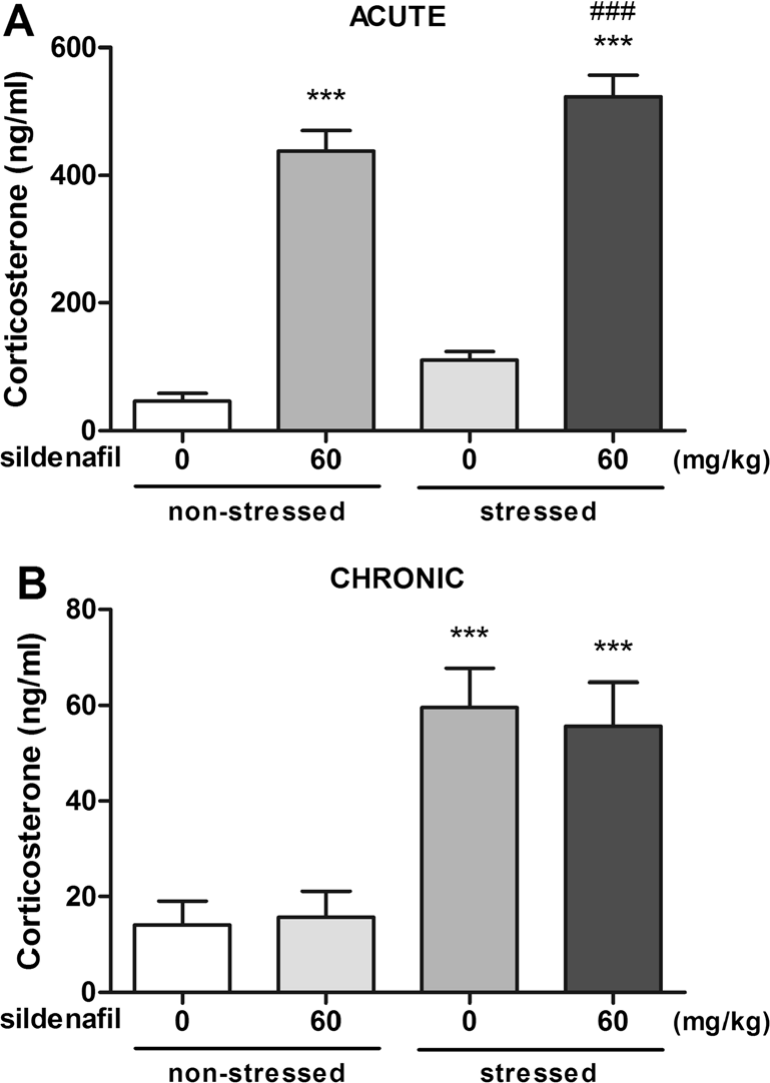
Effect of acute (panel **A**) or subchronic (panel **B**) treatment with sildenafil and acute restraint stress on serum corticosterone concentrations in mice. In acute studies, sildenafil was administered i.p. 30 min before the test. In subchronic studies, sildenafil was administered once daily for 14 consecutive days. Control animals received 1 % Tween 80 + 1 % DMSO. Each experimental group consisted of 9–11 animals. *** *p* < 0.001 as compared to the non-stressed control group; ^###^ *p* < 0.001 as compared to the control stressed group (two-way ANOVA followed by Bonferroni *post hoc* test)

The influence of acute sildenafil treatment on serum corticosterone level in non-stressed and stressed animals is shown in Fig. 4A. A two-way ANOVA revealed a significant effect of acute restraint stress (F(1,37) = 9.40, *p* = 0.004), a significant effect of treatment (F(1,37) = 275.06, *p* < 0.0001) and no interaction between acute restraint stress and sildenafil treatment (F(1,37) = 0.15, *p* = 0.698). Although acute restraint stress increased corticosterone concentration in control animals, a Bonferroni *post hoc* test did not show a statistically significant effect. Acute administration of sildenafil in non-stressed animals caused a marked increase in corticosterone level (*p* < 0.001 vs. the non-stressed control group). In mice subjected to the acute restraint stress, sildenafil caused a further increase in corticosterone concentration (*p* < 0.001 vs. the non-stressed control group and the stressed control group).

The influence of repeated sildenafil treatment on serum corticosterone level in non-stressed and stressed animals is shown in Fig. 4B. A two-way ANOVA revealed a significant effect of acute restraint stress (F(1,37) = 32.70, *p* < 0.0001), no significant effect of treatment (F(1,37) = 0.02, *p* = 0.878) and no interaction between acute restraint stress and sildenafil treatment (F(1,37) = 0.14, *p* = 0.714). A Bonferroni *post hoc* test showed a statistically significant increase in corticosterone level in control mice subjected to the acute restraint stress as well as in stressed mice injected subchronically with sildenafil (*p* < 0.001 vs. the non-stressed control group).

## Discussion

Targeting PDE5 with sildenafil has recently gained attention of some researchers as a new therapeutic approach in the treatment of depression (Matsushita et al. 2012; Tomaz et al. 2014; Wang et al. 2014). The antidepressant-like effect of sildenafil has been assessed almost exclusively in the forced swim test in rodents that remains one of the most widely used tools for screening antidepressants. As the influence of sildenafil on depressive-like behavior in this test is ambiguous, we aimed to provide more data on the effect of sildenafil administered acutely and subchronically in the forced swim test in mice. In the first series of experiments, the antidepressant-like efficacy of sildenafil injected alone in naïve mice was evaluated. In our former study, acute administration of sildenafil at doses of 5–20 mg/kg did not affect animal behavior in the forced swim test in mice (Socała et al. 2012c). In the present study, we showed that sildenafil produced an antidepressant-like effect at a relatively high dose of 60 mg/kg. We expected that repeated treatment would potentiate the antidepressant-like effect of sildenafil. However, after subchronic administration, sildenafil showed poor anti-immobility action, only at the highest dose of 60 mg/kg. In the next series of experiments, the effect of acute and subchronic treatment with sildenafil was investigated in mice with depressive-like behavior induced by reserpine pretreatment or acute restraint stress. Both reserpine and acute immobilization prolonged the duration of immobility in mice. Sildenafil given at doses of 40 and 60 mg/kg reversed the behavioral despair in reserpine-treated as well as in stressed mice. By contrast, subchronic treatment with sildenafil did not produce any significant effects.

The main findings of the present study indicated differential effects of acute and subchronic treatment with sildenafil in the forced swim test in mice. Sildenafil exerted antidepressant-like effects only after acute treatment with high doses that are 3–5 times higher than the equivalent recommended dose for humans (50 mg) and this observation is consistent with findings of Matsushita and co-workers (2012). The lack of antidepressant-like activity after subchronic treatment is rather unexpected as sildenafil has been already reported to have antidepressant-like effect after chronic treatment in mice (Wang et al. 2014). A possible explanation for the discrepancy between our results and results obtained by Wang et al. (2014) is a different scheme of chronic treatments. Wang et al. (2014) reported the antidepressant-like effect of sildenafil (30 mg/kg) injected repeatedly for 21 days with the last injection made 60 min before the forced swim test in mice. In our study, sildenafil was administered for 14 consecutive days and the animals were subjected to the swim session 24 h after the last administration. There was no additional injection on the day of the forced swim test. This treatment schedule was chosen in order to avoid any acute effects of sildenafil treatment and it was based on the observation made by Dadomo and co-workers (2013). In their study, sildenafil was given every other day for 35 days and all behavioral tests were performed in the day without an injection. The obtained results showed that sildenafil administered in this manner increased competitive aggression, environmental and social exploration and reduced anxiety-like behaviors (Dadomo et al. 2013). It seems that after chronic exposure to sildenafil, some behavioral effects should be anticipated ~24 h after the last injection and there was no definite need for the additional injection on the day of the behavioral tests. This manner of administration helps to distinguish between acute and chronic effects of the tested compound. Noteworthy, the anti-immobility effects of antidepressant compounds injected chronically with 24-h break between the last injection and swim session were reported (Doboszewska et al. 2015; Guo and Lu 2014; Nowak et al. 2003).

Several explanations can be proposed to account for the differential effects of acute and repeated sildenafil treatment. Firstly, the lack of antidepressant-like effect after subchronic treatment could have been a result of the activation of negative feedback mechanism within the NO/cGMP/PDE5 signaling pathway. Prolonged inhibition of PDE5 by repeated sildenafil administration leads to the accumulation of cGMP. A high concentration of cGMP could have caused a negative feedback on NO synthase with subsequent reduction of NO level (Hotchkiss et al. 2005; Kyratsas et al. 2013). Nitric oxide plays a prominent role in the pathogenesis of depression because it modulates the release of many neurotransmitters such as serotonin, noradrenaline, dopamine and glutamate (Dhir and Kulkarni 2011). Thus, different changes in NO concentration after acute and repeated sildenafil treatment might have been accountable for different behavioral response in the forced swim test. Of note, differential effects of acute and chronic sildenafil treatment on serotonin and dopamine turnover have already been reported (Kyratsas et al. 2013).

Secondly, a possible withdrawal effect following repeated sildenafil administration should be considered. Although the issue of addiction to sildenafil was not extensively studied, epidemiological studies show that sildenafil is sometimes abused in a recreational fashion. Sildenafil was also reported to exert rewarding properties in the conditioned place preference paradigm in mice (Tahsili-Fahadan et al. 2006). Moreover, sildenafil-treated mice were more aggressive one week following cessation of 4 weeks of treatment (Hotchkiss et al. 2005). Increased immobility time in the forced swim test is one of the behavioral symptoms of withdrawal in rodents. In the present study, the lack of the anti-immobility action after subchronic treatment could have been one of the behavioral effects associated with discontinuation of the treatment. Nevertheless, this is only a speculation and possible addictive effects of sildenafil use should be carefully evaluated in further studies.

Although the forced swim test is the most commonly used animal model for assessing both depressive- and antidepressant-like activity, a range of factors influence the behavior in the forced swim test and complicates the comparison across different studies. These factors include strain of mice, age, gender, individual differences between animals, housing, pretreatment schedule, laboratory conditions, depth of water or exposure to the pre-test swim session (Bogdanova et al. 2013; Petit-Demouliere et al. 2005). Some of these factors could have also contributed to the difference between our results and the results obtained by other groups. For instance, we scored animals only for the last 4 min of the 6-min swim session and no pre-test session was employed in the present study, as it is not recommended for mice (Gardier and Bourin 2001; Petit-Demouliere et al. 2005). In contrast, Matsushita et al. (2012), who reported the antidepressant-like effect of sildenafil after a sub-chronic treatment, scored the immobility time for the last 3 min of the 6-min swim session after the pre-test swim session.

Total brain and serum concentrations of sildenafil after acute and repeated treatment were determined in order to check whether repeated administration of sildenafil might result in accumulation of this compound in the organism. Interestingly, sildenafil was not detectable in the brain tissue 24 h after the termination of a 14-days continuous sildenafil treatment. In serum, sildenafil was still measurable after discontinuation of the treatment but at much lower concentrations than those after an acute administration. These results suggest that the lack of anti-immobility action of sildenafil after subchronic treatment was related to the fact that the drug was eliminated from brain within 24 h following the last injection. In clinical practice, antidepressants have a delayed onset of action and chronic treatment is required. Interestingly, in the forced swim test the effectiveness of acute treatment with antidepressant agents is a well-known phenomenon and chronic treatment usually increase the antidepressant potential of the tested compounds (Petit-Demouliere et al. 2005). It is presumed that repeated treatment with antidepressant drugs leads to long-term adaptive changes, which account for the antidepressant effect. These changes include receptor desensitization, alteration in cell signaling and synaptic plasticity, stimulation of neurogenesis, or changes in gene expression (Millan 2006). Therefore, there is a need for more studies assessing the above-mentioned adaptive changes after chronic exposure to sildenafil.

It is widely known that there is a close relationship between stress, the hypothalamic–pituitary–adrenocortical (HPA) axis and depression. The HPA axis is activated upon exposure to stress situation, which triggers the release of cortisol (in humans) or corticosterone (in mice) from the adrenal cortex (Sturm et al. 2015). In the present study, we also aimed to evaluate the influence of sildenafil on corticosterone release in non-stressed and stressed mice. In studies assessing acute effects of sildenafil, acute restraint stress caused a ~ 3-fold increase in serum corticosterone level. Unexpectedly, sildenafil administration produced a ~ 9.5- and 11.5-fold increase in serum corticosterone in non-stressed and stressed animals, respectively. To the best of our knowledge, there is no data on the impact of sildenafil on corticosterone release. Inhibition of PDE5 and subsequent accumulation of cGMP may affect the HPA axis at all levels and increase the secretion of corticosterone. The mechanism underlying the ability of sildenafil to increase corticosterone secretion remains to be determined. Of note, there is one study, in which cGMP injected directly into the hypothalamus caused a marked increase in serum corticosterone level in rats (Endroczi 1980). In studies assessing the effect of sildenafil injected repeatedly, sildenafil did not cause any changes in corticosterone concentrations. This could be explained by the dramatic changes in sildenafil concentrations in serum and brain within 24 h after the termination of the subchronic treatment.

Compounds that increase general locomotor activity can provide false positive results in the forced swim test. Since sildenafil did not affect locomotor activity in the present study, its effects in the forced swim test were not related to alterations in locomotion.

In summary, the present findings demonstrate that sildenafil, at a relatively high dose, exerts better antidepressant-like activity in the forced swim test after acute than repeated treatment. Moreover, acute but not subchronic treatment with sildenafil reversed the reserpine- and stress-induced behavioral despair in mice. The obtained results did not confirm previous suggestions (Matsushita et al. 2012; Socała et al. 2012c; Tomaz et al. 2014; Wang et al. 2014) that sildenafil would be an effective therapy for depression, especially given the concern raised by the acute effect of sildenafil on corticosterone release.

## Abbreviations

cGMP: cyclic guanosine 3´,5´-monophosphate
i.p.: intraperitoneally
IS: internal standard
LPS: lipopolysaccharide
NO: nitric oxide
PDE5: phosphodiesterase type 5
SEM: standard error of the mean
